# Functional characterization of cellulases identified from the cow rumen fungus ***Neocallimastix patriciarum *** W5 by transcriptomic and secretomic analyses

**DOI:** 10.1186/1754-6834-4-24

**Published:** 2011-08-17

**Authors:** Tzi-Yuan Wang, Hsin-Liang Chen, Mei-Yeh J Lu, Yo-Chia Chen, Huang-Mo Sung, Chi-Tang Mao, Hsing-Yi Cho, Huei-Mien Ke, Teh-Yang Hwa, Sz-Kai Ruan, Kuo-Yen Hung, Chih-Kuan Chen, Jeng-Yi Li, Yueh-Chin Wu, Yu-Hsiang Chen, Shao-Pei Chou, Ya-Wen Tsai, Te-Chin Chu, Chun-Chieh A Shih, Wen-Hsiung Li, Ming-Che Shih

**Affiliations:** 1 Biodiversity Research Center, Academia Sinica, Taipei 115, Taiwan; 2 Genomics Research Center, Academia Sinica, Taipei 115, Taiwan; 3 Graduate Institute of Biotechnology, National Pingtung University of Science & Technology, Neipu Hsiang, Pingtung 91201, Taiwan; 4 Department of Life Sciences, National Cheng Kung University, Tainan 701, Taiwan; 5 Molecular and Biological Agricultural Sciences Program, Taiwan International Graduate Program, National Chung-Hsing University - Academia Sinica, Taipei 115, Taiwan; 6 Graduate Institute of Biotechnology, National Chung-Hsing University, Taichung 402, Taiwan; 7 Agricultural Biotechnology Research Center, Academia Sinica, Taipei 115, Taiwan; 8 PhD Program in Microbial Genomics, National Chung Hsing University, Taichung 402, Taiwan; 9 Department of Life Sciences, National Taiwan University, Taipei 106, Taiwan; 10 Institute of Information Science, Academia Sinica, Taipei 115, Taiwan; 11 Department of Computer Science and Information Engineering, National Taiwan Normal University, Taipei 116, Taiwan; 12 Biotechnology Center, National Chung-Hsing University, Taichung 402, Taiwan; 13 Department of Ecology and Evolution, University of Chicago, Chicago, IL 60637, USA

**Keywords:** anaerobic fungi, biomass, rice straw, sugarcane, napiergrass, *GH*, next-generation sequencing

## Abstract

**Background:**

*Neocallimastix patriciarum* is one of the common anaerobic fungi in the digestive tracts of ruminants that can actively digest cellulosic materials, and its cellulases have great potential for hydrolyzing cellulosic feedstocks. Due to the difficulty in culture and lack of a genome database, it is not easy to gain a global understanding of the glycosyl hydrolases (*GHs*) produced by this anaerobic fungus.

**Results:**

We have developed an efficient platform that uses a combination of transcriptomic and proteomic approaches to *N. patriciarum *to accelerate gene identification, enzyme classification and application in rice straw degradation. By conducting complementary studies of transcriptome (Roche 454 GS and Illumina GA IIx) and secretome (ESI-Trap LC-MS/MS), we identified 219 putative *GH *contigs and classified them into 25 *GH* families. The secretome analysis identified four major enzymes involved in rice straw degradation: β-glucosidase, endo-1,4-β-xylanase, xylanase B and Cel48A exoglucanase. From the sequences of assembled contigs, we cloned 19 putative cellulase genes, including the *GH1*, *GH3*, *GH5*, *GH6*, *GH9*, *GH18*, *GH43 *and *GH48 *gene families, which were highly expressed in *N. patriciarum *cultures grown on different feedstocks.

**Conclusions:**

These *GH *genes were expressed in Pichia pastoris and/or Saccharomyces cerevisiae for functional characterization. At least five novel cellulases displayed cellulytic activity for glucose production. One β-glucosidase (W5-16143) and one exocellulase (W5-CAT26) showed strong activities and could potentially be developed into commercial enzymes.

## Background

Cellulosic ethanol produced by microbial fermentation from feedstocks has been proposed to replace fossil fuels in transportation. A key step in cellulosic ethanol production is to break down cellulose into glucose and hemicellulose into xylose, which can subsequently be converted into ethanol by fermentative microbes. Therefore, finding efficient cellulases is important to bioethanol production, as well as for hydrolyzing feedstocks into sugars in general. *Neocallimastix *species is one of the major anaerobic fungi in the rumen of water buffalo capable of efficiently digesting cellulosic biomass [1-4]. Such anaerobic fungi are potential sources for highly active cellulolytic enzymes that are useful for cellulose hydrolysis [5-7]. Plant cell wall degrading enzymes from rumen fungi such as *Neocallimastix patriciarum *may be used for the production of industrial materials from plant biomass. These enzymes may also improve the fiber properties of cotton for manufacturing or clothes. The simple sugars which retain the chemical energy of lignocellulose are easily separated from the digestion products and more readily usable for animal or human food or for the production of chemicals and biofuels [7]. Pai *et al*. [8] reported the cloning from *N. patriciarum *of a bifunctional xylanolytic enzyme with acetylxylan esterase and xylanase activities. Interestingly, this enzyme contains a double-dockerin domain, suggesting that it is a cellulosomal component and may bind tightly to the cellulosome [8].

Microbial genomes often contain a substantial number of glycosyl hydrolase (*GH*) genes, many of which respond to different carbon sources. There are many cellulases, such as xylanase and glycosidase, identified in rumen fungi; however, only a fraction of these exocellulases and β-glucosidases (BGLUs) have shown high enzymatic activities [9-18]. The concentration of extracellular cellulase proteins of *N. patriciarum *W5 detected in our laboratory was about 138.2 to 193.7 mg/L, of which 30% showed xylanase activity. However, there is no feasible method for long-term preservation of rumen fungal cultures. Anaerobic fungal isolates have to be transferred every two to six days to maintain their activity [19]. To overcome this limitation, previous studies using traditional genetic screening approaches identified several cellulase-related genes from *N. patriciarum *and expressed them in *Escherichia coli *[8,20-28]. For example, the cellulobiohydrolase of *N. patriciarum *showed a 1,000 times greater specific activity than that of the cellulobiohydrolase of *Trichoderma reesei *[23]. Such higher cellulase activity can significantly reduce the cost of the desaccharification during cellulosic ethanol production. However, the conventional methods for identifying microbial cellulase genes through purification of cellulosic proteins and/or protein sequence-based cDNA cloning were tedious and time-consuming.

Recent advances in genomics, transcriptomics and proteomics technologies make hunting for cellulase genes much more efficient. Proteomics analysis using liquid chromatography coupled with tandem mass spectrometry (LC-MS/MS) can be used to develop native protein databases that depict the nature and levels of proteins expressed in microbes [29,30]. Without a closely related protein sequence database, however, one cannot survey the novel microbial protein profile comprehensively, and full-length sequence information of these proteins for further characterization is required, especially for enzymes present as gene families. In contrast, high-throughput next-generation sequencing can provide abundant cDNA information by using long-read transcriptome sequencing by GS FLX Sequencer (454 Life Sciences/Roche, Branford, CT, USA), and subsequently protein sequences can be derived and used as references for proteomic mapping, enabling the functional profiling of protein diversity and quantification. In parallel, the levels of gene expression can be examined by short-read deep sequencing using Genome Analyzer IIx (Illumina Inc., San Diego, CA, USA). Therefore, proteomic and transcriptomic data can offer complementary information in the hunt for valuable cellulase genes. Here we demonstrate how a combination of omics approaches helped us to identify glycosyl hydrolase (*GH*) gene families and functional proteins from a nonmodel organism, *N. patriciarum *W5 strain.

In this report, we describe the characterization of several cellulosic genes from the newly isolated *N. patriciarum *W5 strain by complementary secretome and transcriptome analyses. We cultured the W5 strain in different cellulosic substrates such as rice straw, napiergrass and sugarcane powder to enrich cellulase protein expression, and we purified extracellular proteins exhibiting dominant cellulase activity by both enzymatic assays and zymograms. These major cellulase proteins were then identified by LC-MS/MS analysis and online *GH *family database surveys. The secretome data successfully hit 25 *GH *family protein sequences. In parallel, the enriched transcriptomes were sequenced for global gene identification. By combining these two omics data, we successfully identified 19 major extracellular cellulase genes of *N. patriciarum *and cloned them in two yeast expression systems for functional characterization.

## Results and Discussion

### Extracellular cellulases of ***N. patriciarum *** W5

**Figure 1:**
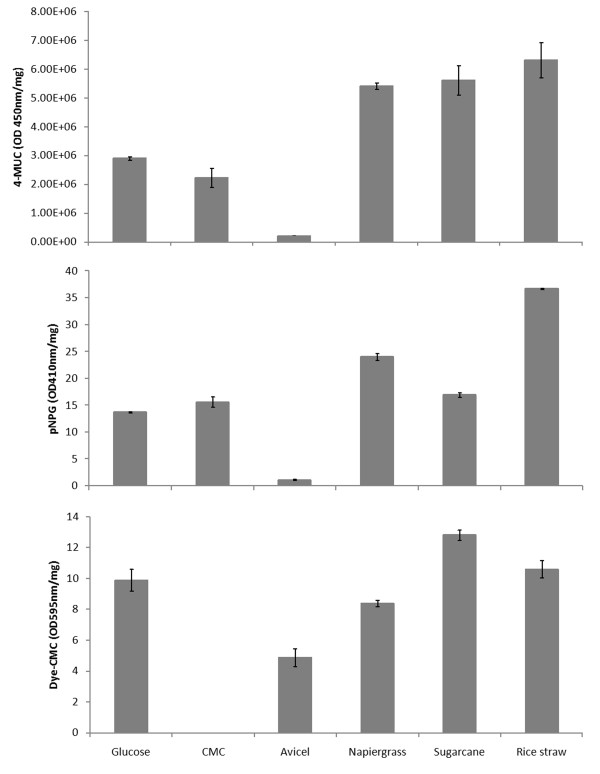
**Total cellulase activity of four-day culture of W5 strain in response to glucose, two artificial celluloses (CMC and avicel) and three feedstocks (napiergrass, sugarcane and rice straw)**.

To stimulate the *N. patriciarum *W5 strain to produce cellulosic enzymes, we used powders of rice straw, napiergrass and sugarcane bagasse. All three cultures had the highest total cellulase activities on day 3 or 4 during the time course assayed by 4-methylumbelliferyl-β-cellobiose (4-MUC) analysis (data not shown). To examine the different cellulase enzymatic activities, we collected the total extracellular proteins (supernatant) at day 4 for further analyses. We assayed the cellulase activities using 4-MUC, *p*-nitrophenyl-β-D-glucopyranoside (*p*NPG) and dye CM-cellulose (dye-CMC) as substrates and performed cross-analyses to determine the levels of exoglucanase (EXG), endoglucanase (EG) and BGLU activities in the total extracellular protein extracts (see Methods). We used the following rules to examine EXG, EG and BGLU activities: 4-MUC assays for broad quantitative assays of cellulase activity, dye-CMC assays for EG activity and *p*NPG assays for BGLU activity. If both dye-CMC and *p*NPG activities were low, the 4-MUC activity was taken to represent the exocellulase activity. Our results showed that the cultures grown in rice straw produced the highest extracellular EXG and BGLU activities, and the cultures grown in sugarcane bagasse exhibited the highest endocellulase activity (Figure 1).

### Cellulolytic enzyme induction in ***N. patriciarum *** W5 by different substrates

The exo-type cellulolytic activities increased with time to the highest level on the third and fourth days after *N. patriciarum *W5 was induced by different carbon sources (data not shown). The fourth-day culture broth was centrifuged, and the crude enzyme solution was prepared by collecting the supernatant for further analyses of enzyme activities and secretome. The cellulolytic activities of crude enzymes were determined using 4-MUC, dye-CMC and *p*NPG as substrates. As shown in Figure 1, feedstock of plant origins such as napiergrass, sugarcane bagasse and rice straw demonstrate a better induction effect to the cellulase production of *N. patriciarum *W5 than commercial substrates. The highest activities of EXG and BGLU were found in the broth supplemented by rice straw, whereas the crude enzyme induced by sugarcane bagasse exhibited the highest EG among tested substrates. Rice straw was able to induce the best activities of cellulytic enzymes and was selected as an inducer for the following analyses.

### Secretome analysis of ***N. patriciarum *** W5

**Figure 2:**
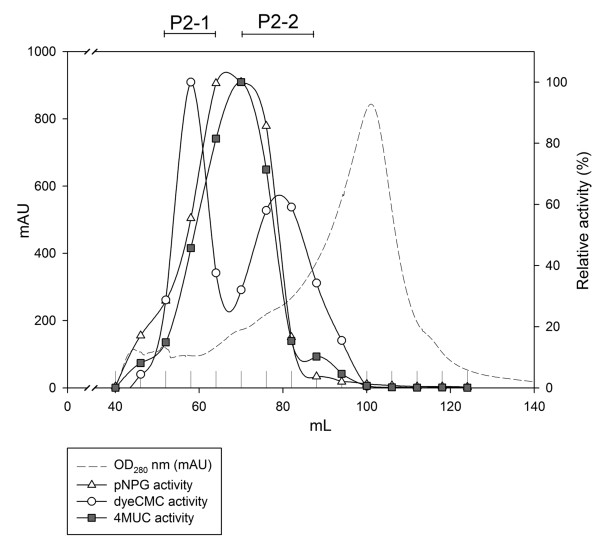
**Sample fractions (in milliliters) and relative cellulase activity (%) after QFF P2-Superdex 200 purification**.

To gather more information on cellulase proteins and eliminate interference of other proteins, we enriched and purified the extracellular cellulase proteins from W5 cultures grown on rice straw-containing medium as shown in Figure 1. To purify the extracellular cellulases, the proteins in condensed supernatants were first subjected to anion exchange chromatography (HiPrep™ 16/10 QFF, GE Healthcare Bio-Sciences AB, Uppsala, Sweden) and then by size exclusion (HiLoad™ Superdex™ 200 prep grade Column, GE Healthcare Bio-Sciences AB, Uppsala, Sweden) at 4°C. After these two steps, the specific activities for 4-MUC, *p*NPG and dye-CMC were 78.97, 2.17 and 1.15 U/mg, respectively (Table 1). Accordingly, the P2 fraction gathered most purified cellulase proteins by 1.9- to 4.7-fold (purification factor) compared to the supernatants. The purified cellulases were further fractionated into two major groups with different protein sizes (P2-1 and P2-2 in Figure 2). The dye-CMC assay indicated that these two major fractions both contained EG activity, and the 4-MUC and *p*NPG assays indicated that both fractions contained EXG as well as BGLU activity. These data suggest that there are at least two sizes of EG and one size each of EXG and BGLU.

**Table 1:** Purified cellulase activity in culture with rice straw^a^

	Culture supernatant	Concentration + buffer exchange	QFF anion exchange	S200 gel filtration P1	S200 gel filtration P2
Fraction volume, mL	500	3	4	1	1
Total protein, mg	27.81	8.97	2.24	1.39	0.23
4-MUC					
Total activity, U	470.71	196.33	67.21	26.17	17.94
Specific activity, U/mg	16.93	21.90	29.95	18.89	78.97
Yield, %	100%	42%	14%	6%	4%
Purification factor	1.0	1.3	1.8	1.1	4.7
*p*NPG					
Total activity, U	22.54	5.57	1.91	0.86	0.49
Specific activity, U/mg	0.81	0.62	0.85	0.62	2.17
Yield, %	100%	25%	8%	4%	2%
Purification factor	1.0	0.8	1.1	0.8	2.7
dye-CMC					
Total activity, U	16.79	3.36	0.78	0.07	0.26
Specific activity, U/mg	0.60	0.38	0.35	0.05	1.15
Yield, %	100%	20%	5%	0%	2%
Purification factor	1.0	0.6	0.6	0.1	1.9

^a^4-MUC: 4-methylumbelliferyl-β-cellobiose; dye-CMC: dye CM-cellulose; *p*NPG: *p*-nitrophenyl-β-D-glucopyranoside;

**Figure 3:**
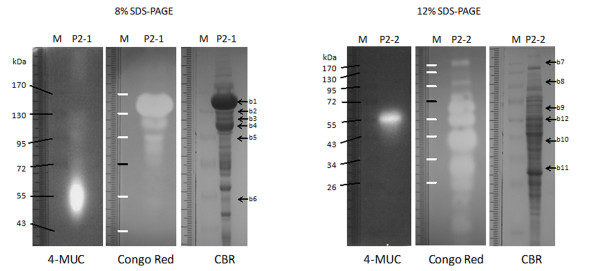
**Zymogram assay of the second batch culture**. Fractions P2-1 and P2-2 in Figure 2 corresponding to 52 to 61 mL and 64 to 73 mL were pooled and then separated on 8% and 12% SDS-PAGE-based zymogram assays, respectively. Twelve bands were recovered and analyzed for possible glucanase sequences using ESI-Trap LC-MS/MS.

To demonstrate that these partially purified proteins were indeed cellulases, we conducted zymogram analysis by CMC blot assay (Figure 3). Our results show that the *N. patriciarum *cultures were induced to express various sizes of putative EG/BGLU (ranging from 20 to 170 kDa), but only one major size of putative EXG/BGLU (around 55 kDa) was found in the 4-MUC zymogram assay. These data were consistent with the fractionation data. Based on the zymogram blot analysis, a total of 17 bands in PAGE were selected from three different batch cultures and subsequently analyzed by ESI-Trap LC-MS/MS and a BLAST homology search using the MASCOT online database (see Additional file 1). The peptide sequences generated from MS/MS spectra were successfully mapped to 24 *GH *genes, including five endocellulases, twelve exocellulases and seven BGLUs from three genera of rumen fungi: *Neocallimastix*, *Orpinomyces *and *Piromyces*. The mass data showed that the protein sequences of the Cel48A, Cel9A and BGLU precursors as well as BGLU Cel1C from *Piromyces *sp. E2 were frequently mapped by 25, 12, 43 and 28 different peptides, respectively. In addition, another BGLU from *Orpinomyces *sp. PC-2 was also highly mapped by 27 different peptides, and there were 4 endocellulases found in the mass data. The most significant hit, a GUNB_NEOPA EG B precursor, was mapped by seven different peptide sequences. These results suggest that at the time of harvesting, the culture might have a higher abundance of BGLUs than other cellulases. However, there were a few peptides matched with exocellulases, possibly due to the low abundance proteins and/or insufficient rumen fungi online protein sequences. The transcriptome data could alleviate such insufficiency.

### Transcriptomic analysis of ***N. patriciarum *** W5

To induce the expression of as many kinds of proteins involved in biomass degradation as possible, we enriched the transcriptome by culturing the W5 strain separately in defined medium with different celluloses (CMC, avicel and cellobiose) and feedstocks (rice straw, napiergrass and sugarcane). To prepare a sample for transcriptome sequencing, equal amounts of total RNA from independent batch cultures were pooled together. The combined RNA sample was first converted into double-stranded cDNA, then fragmented by nebulization, made into a shotgun library and subjected to Roche 454 GS FLX sequencing.

A total of 526,516 raw reads were generated from sequencing of this transcriptome. Nucleotide content analysis indicated that *N. patriciarum *W5 has high AT content (63%) (Table 2), which is consistent with its genome sequence (data not shown). To obtain the first draft of expressed sequences, the raw reads were assembled using Newbler version 1 software (454/Roche), leading to the initial contig data set. Sequences longer than 500 bp were selected for BLAST searches of the National Center for Biotechnology Information (NCBI) database and scored for the top hits with similarity to known cellulase genes. The candidates were then subjected to cDNA cloning.

**Table 2:** Summary of transcriptome analyses^a^

Transcriptome analyses	W5 cDNA
Roche 454 GS FLX	
Run cycles Total number of reads Total read length, Mb	1 526,516 111
Average read length, bp	210.2
Illumina GA II Paired-End	
Read length, bp	40 + 40
Run lanes	1
Total number of reads Total read length, Mb	14,802,234 1,184
GC content	37.1%
Contig assembly	
Contig numbers	20,232
Total contig lengths, Mb	9.0
Average contig length, bp	447.2
Largest contig length, bp	4,397
Gene prediction, length > 500 bp	
Number of ORFs	2,284
Number of domains predicted in ORFs	9,720
Average ORF length, bp	846.9
Maximum RPKM^b ^value	31,647.2
Number of *GH*-like contigs	219

^a^GC: the nucleotide G+C % ; *GH*: glycosyl hydrolase; ORF: open reading frame; RPKM: Reads Per Kilobase of exon model per Million mapped reads. ^b^RPKM is a method for estimating relative expression levels of a transcriptome by normalizing short-read expressed tags from deep sequencing over ORF length and total reads (RPKM) [35].

**Figure 4:**
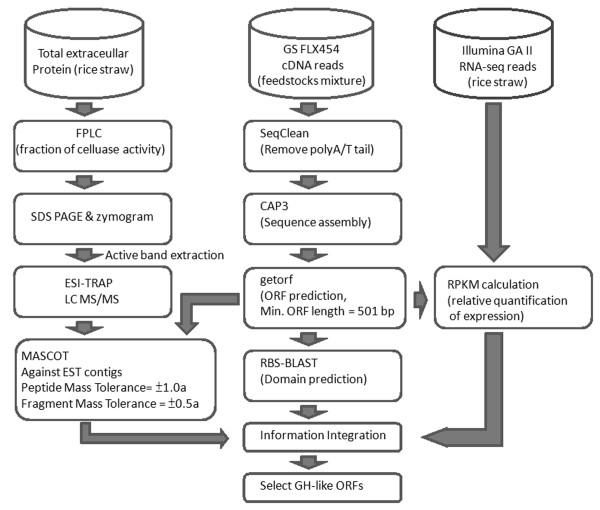
**Flowchart showing the transcriptome and secretome analysis pipeline**.

To obtain an EST data set with higher assembly accuracy, the 454 raw reads were first trimmed by the SeqClean program (http://compbio.dfci.harvard.edu/tgi/software/) to remove poly(A/T) tails. The preprocessed reads were then assembled into 20,232 unique contigs using CAP3 software [31]. As shown in Figure 4, the resulting EST contigs were used for six-frame open reading frame (ORF) prediction by GETORF software [32]. A total of 2,284 ORFs greater than 500 bp in length were selected and translated into protein sequences as an input for domain prediction and for a LC-MS/MS in-house database. Domain prediction was conducted by conducting a RPS-BLAST search against the NCBI Conserved Domain Database (CDD) using default parameters [33]. Contigs related to sugar metabolism and cellulose degradation were selected based on domain prediction results using keywords such as "gly," "*GH*" and "ase." The top-scoring NCBI sequences were collected, totaling 153 known annotations from the 288 unique *GH*-like contigs (497 ORFs), including cellulases, hemicellulases, lipases, chitinases and cellulosome dockerin proteins. Among those, 219 contigs could be further classified into 25 putative *GH *families (Table 3 and Additional file 2). The top four largest classes (contig numbers > 20) of putative *GH *families were *GH6 *(15%), *GH10 *(9.5%), *GH5 *(9.1%) and *GH43 *(9.1%). Based on the Carbohydrate-Active EnZymes (CAZy) database (http://www.cazy.org/), the *GH6 *family is mainly composed of EG (EC 3.2.1.4) and cellobiohydrolase (EC 3.2.1.91), whereas the *GH5*, *GH10 *and *GH43 *families are mainly composed of xylanases [34]. Interestingly, the cellulases induced by these feedstocks are more complex than our expectation, as revealed by their high diversity.

**Table 3:** Expressed cellulase- and hemicellulose-degrading enzymes of *Neocallimastix patriciarum *arranged by *GH *family^a^

	Cellulase										Hemicellulase											
			
Putative *GH *family	1	3	5	6	7	9	12	45	48	61	10	11	26	29	43	51	53	54	62	67	74	95
*Neocallimastix patriciarum *W5	7	10	20^b^	33^b^		12^b^		14^b^	12^b^		21^b^	15^b^	4^b^		20^b^		1					
*Neurospora crassa *OR74A	1	9	7	3	5		1	1		14	4	2	1		7	1	1	1^b^		1^b^	1	
*Magnaporthe grisea *70-15	2	19	13	3	6^b^		3	1		17	5	5		4^b^	19	3	1	1^b^	3^b^	1^b^	1	1
*Aspergillus nidulans *FGSC A4	3	20	15	2	3		1	1		9	3	2	3		15	2	1	1^b^	2	1^b^	2^b^	3^b^
*Aspergillus niger *CBS 513.88	3	17	10	2	2		4^b^			7	1	4	1	1	10	4^b^	2^b^	1^b^	1	1^b^	1	2
*Aspergillus oryzae *RIB40	3	23^b^	14	1	3		4^b^			8	4	4	1		20^b^	3	1	1^b^	2	1^b^		3^b^
*Leptosphaeria maculans *v23.1.3	3	13	15	3	3		3	2		20^b^	3	2	1	1	11	3	1		1	1^b^		1
*Trichoderma reesei*^c^	2	13	11	1	2		2	1		2	1	3			2				1	1^b^		

^a^*GH*: glycosyl hydrolase; CAZy: Carbohydrate-Active enZYmes database. ^b^Highest gene number among fungi from JGI database and CAZy annotation. ^c^Adapted from JGI database.

Our comparative fungi *GH *family gene analysis showed that *N. patriciarum *W5 has a greater number of cellulases and hemicellulases than in the other fungi studied, 108 and 61, respectively (Table 3). *T. reesei *only has 34 cellulases and 8 hemicellulases, and *Aspergillus niger *has 45 cellulases and 29 hemicellulases. In the non-cellulose-degrading yeasts such as *Kluyveromyces lactis *and *Yarrowia lipolytica*, only a fraction of *GH3 *and *GH5 *family genes could be found in their genome sequences (data not shown). Our transcriptome analysis showed that *N. patriciarum *W5 utilized several common *GH *gene families, such as *GH1*, *GH5*, *GH6*, *GH10*, *GH11 *and *GH43*, and also specific gene families, such as *GH9 *and *GH48*, for degrading the cellulose of rice straw (Table 3). These observations suggest that a combination of several cellulases may be required to hydrolyze rice straw more efficiently. The diversity and specificity of *GH *gene families could provide synthetic biologists with the opportunity to build up a yeast host with these major *GH *genes for consolidated bioprocess (CBP) and/or coculture processing.

To investigate the quantitative gene expression in W5 cultured with rice straw, independent transcriptome sequencing was performed using the Illumina Genome Analyzer IIx for greater sequencing depth and throughput. A total of 14.8 million of the Illumina Genome Analyzer IIx paired-end reads (Table 2) were mapped to the EST contig data set assembled by CAP3 to calculate Reads Per Kilobase of exon model per Million mapped reads (RPKM) for all contigs [35]. The quantification analyses (Tables 4 and 5) indicate the relative expression levels of the *GH*-like contigs identified in the secretome and those from sequencing and cloning, respectively.

**Table 4:** Forty unique *GH*-like contig hits by BLAST analysis of secretome mass data against transcriptome assembly^a^

Contig name	Putative *GH *family	Gene length	RPKM^b^ value	Accession number	Enzyme annotation [species name]	Mass spot hits (a: first batch; b: second batch; c: third batch) (number: band ID on SDS-PAGE)
Contig18827	*GH13*	891	105.6	EEQ33998.1	1,4-α-glucan branching enzyme [*Microsporum canis*]	b9
Contig148	*GH13*	819	19.8	XP_963252.2	1,4-α-glucan branching enzyme [*Neurospora crassa*]	b9
Contig1907	*GH1*	882	757.2	CAC34952.1^c^	β-glucosidase [*Piromyces sp*. E2]	a3, a10, b4, b5, b6, b8, b9, b12
Contig11849	*GH3*	2,064	29.0	ZP_04746179.1	β-glucosidase [*Roseburia intestinalis*]	b5
Contig4463	*GH1*	522	2,290.4	AAP30745.1^c^	β-glucosidase Cel1C [*Piromyces sp*. E2]	a11, b2, b4, b5, b10, b12
Contig16557	*GH1*	1,959	1,612.4	AAP30745.1^c^	β-glucosidase Cel1C [*Piromyces sp*. E2]	a3, a10, a11, b1, b2, b4, b5, b8, b9, b10, b12
Contig7663	*GH1*	1,959	1,096.6	AAP30745.1 ^c^	β-glucosidase Cel1C [*Piromyces sp*. E2]	a3, a10, a11, b1, b2, b4, b5, b8, b9, b10, b12
Contig19120	*GH1*	1,548	166.5	AAP30745.1 ^c^	β-glucosidase Cel1C [*Piromyces sp*. E2]	b1, b2, b5, b6
Contig8008	*GH3*	2,277	408.1	AAO41704.1 ^c^	β-glucosidase precursor [*Piromyces sp*. E2]	b2, b3, b4, b5, b6, b8, b12
Contig2687	*GH6*	552	241.0	AAR08200.1	CelA [*Neocallimastix frontalis*]	a11
Contig6374	*GH6*	1,854	24.7	AAM94167.1 ^c^	Cellulosomal exoglucanase Cel6A [*Piromyces equi*]	b5
Contig12342	*GH43*	1,653	664.5	ZP_06201238.1	Conserved hypothetical protein [*Bacteroides *sp. D20]	b12
Contig19148	*GH10*	519	7.8	YP_001181173.1	Endo-1,4-β-xylanase [*Caldicellulosiruptor saccharolyticus*]	b4
Contig1253	*GH43*	2,103	236.9	ACZ98594.1	Endo-1,4-β-xylanase [*Cellulosilyticum ruminicola*]	a11
Contig15284	*GH11*	1,137	341.8	ACL68347.1	Endo-1,4-β-xylanase [*Neocallimastix patriciarum*]	b5
Contig8371	*GH11*	2,412	158.3	ACL68347.1	Endo-1,4-β-xylanase [*Neocallimastix patriciarum*]	b3, b9
Contig19624	*GH45*	696	60.0	CAB92325.1 ^c^	Endoglucanase 45A [*Piromyces equi*]	b3
Contig14117	*GH11*	1,017	159.8	CAA57820.1	Endoxylanase [*Neocallimastix frontalis*]	b9
Contig18110	*GH11*	879	32.3	CAA57820.1	Endoxylanase [*Neocallimastix frontalis*]	b9
Contig10964	*GH18*	687	82.7	YP_001643260.1	GH family protein [*Bacillus weihenstephanensis*]	b4, b8
Contig6246	*GH31*	1,932	232.1	XP_002438844.1	Hypothetical protein [*Sorghum bicolor*]	b2
Contig13446	*GH31*	3,237	301.4	XP_002109215.1	Hypothetical protein [*Trichoplax adhaerens*]	a4
Contig2828	*GH4*	948	9,936.5	CAA76361.1	Malate dehydrogenase [*Piromyces sp*. E2]	b8
Contig9297	*GH10*	993	984.7	AAB30669.1	Xylanase B; XYLB [*Neocallimastix patriciarum*]	b2, b3, b4, b5, b10
Contig13211	*GH10*	819	352.0	AAB30669.1	Xylanase B; XYLB [*Neocallimastix patriciarum*]	b2, b3, b5
Contig13165	*GH10*	990	205.1	AAB30669.1	Xylanase B; XYLB [*Neocallimastix patriciarum*]	b3, b5, b10
Contig8421	*GH10*	1,998	138.5	AAB30669.1	Xylanase B; XYLB [*Neocallimastix patriciarum*]	b9
Contig2430	*GH10*	1,098	132.9	AAB30669.1	Xylanase B; XYLB [*Neocallimastix patriciarum*]	b10, b12
Contig3792	*GH10*	927	5.1	AAB30669.1	Xylanase B; XYLB [*Neocallimastix patriciarum*]	a5
Contig11636	*GH43*	510	966.2	BAC75546.1	Xylosidase [*Penicillium herquei*]	a1, c11
Contig8146	*GH1*	1,986	2,290.4	AAD45834.1 ^c^	β-glucosidase [*Orpinomyces *sp. PC-2]	b2, b12
Contig13432	*GH1*	723	33.0	AAD45834.1 ^c^	β-glucosidase [*Orpinomyces sp*. PC-2]	a8, a10, b5, b6
Contig13654	*GH48*	2,271	6,167.8	AAN76734.1 ^c^	Cellulase Cel48A precursor [*Piromyces sp*. E2]	a2, a3, a10, a11, b10, b12
Contig4845	*GH48*	2,286	362.2	AAN76734.1 ^c^	Cellulase Cel48A precursor [*Piromyces sp*. E2]	b10
Contig6576	*GH48*	1,896	351.4	AAN76734.1 ^c^	Cellulase Cel48A precursor [*Piromyces sp*. E2]	a11
Contig69	*GH48*	1,179	141.9	AAN76734.1 ^c^	Cellulase Cel48A precursor [*Piromyces sp*. E2]	a2, a3, b12
Contig2523	*GH48*	1,596	86.8	AAN76735.1 ^c^	Cellulase Cel48A precursor [*Piromyces equi*]	a2, a11, b12
Contig6878	*GH9*	1,032	285.9	AAM81967.1	Cellulase Cel9A precursor [*Piromyces sp*. E2]	b9
Contig8960	*GH9*	2,037	154.1	AAM81967.1	Cellulase Cel9A precursor [*Piromyces sp*. E2]	b9
Contig7007	*GH11*	1,131	479.8	Q12667	Endo-1,4-β-xylanase A	b2, b3, b4, b5

^a^*GH*: glycosyl hydrolase; RPKM: Reads Per Kilobase of exon model per million mapped reads. ^b^RPKM is a method for estimating relative expression levels of a transcriptome by normalizing short-read expressed tags from deep sequencing over ORF length and total reads (RPKM) [35]. ^c^Online protein sequences hits by using only ESI-Trap LC-MS/MS data against MASCOT online database.

**Table 5:** Nineteen *GH *family genes selected for evaluation of contig assembly and for cellulase expression in yeasts^a^

Cloning sequence ID	Contig ID	Contig length	ORF length	*GH *family	CBM domain	Signal peptide^b^	RPKM value	ESI-Trap LC-MS/MS	Domain prediction^c^	Enzyme activity assay^d^	
										***P. pastoris***	***S. cerevisia***

W5-CAT13	Contig8146	2,691	1,986	*GH1*	-	Y	2,290.4	Y	β-glu	β-glu (S, P)^e^	No activity
W5-16143	Contig4878	1,875	1,815	*GH3*	-	Y	159.8	Y	β-glu	β-glu (S, P)^e^	β-glu (P)^f^
W5-celD^g^	AF053363	3,949	1,383	*GH5*	CBM 10	Y	-	Y	EG + Xyl	EG (S, P)^f^	NA
W5C-P	Contig12293	1,392	1,392	*GH5*	CBM 10	N	405.7		EG	NA	NA
W5-CAT5	Contig8755	1,582	1,479	*GH6*	CBM 10	Y	2,735.9		EXG	No activity	No activity
W5-CAT6-9	Contig8363	1,540	1,497	*GH6*	CBM 10	Y	203.2		EXG	EXG (S, P)^f^	EXG (P)^f^
W5-20147	Contig20147	1,981	1,503	*GH6*	CBM 10	Y	3,576.0		EXG	No activity	No activity
W5C-O	Contig19058	1,449	1,197	*GH6*	CBM 1	Y	1,712.7		EXG	NA	NA
W5-16271	Contig18112	1,754	1,389	*GH6*	CBM 10	Y	760.1		EG + EXG	No activity	No activity
W5-01055	Contig13874	3,935	3,858	*GH6*	CBM 10	N	239.8		EXG	No activity	No activity
W5-10151-39	Contig9299	1,900	1,473	*GH6*	CBM 10	Y	4,397.8		EXG	EXG (S, P)^f^	EXG (P)^f^
W5-10151-38	Contig9839	1,415	1,341	*GH6*	CBM 10	Y	414.1		EXG	EXG (S, P)^f^	NA
W5-10151-5	Contig15588	1,777	1,461	*GH6*	CBM 10	Y	2,727.9		EXG	EXG (S, P)^f^	NA
W5-10151-6	Contig10151	1,852	1,494	*GH6*	CBM 10	Y	654.6		EXG	EXG (S, P)^f^	NA
W5-00992	Contig8960	2,042	2,037	*GH9*	CBM 10	Y	154.1	Y	EG	EG (S, P)^e^	EG (P)^f^
W5-14806	Contig10733	2,509	2,355	*GH9*	CBM 10	Y	165.6		EG	No activity	No activity
W5-CAT24	Contig4514	1,575	1,116	*GH18*	CBM 10	N	88.1	Y	Chitinase	NA	NA
W5-CAT7	Contig19103	2,619	2,262	*GH43*	CBM 6, CBM 10	N	747.4	Y	Xyl	No activity	NA
W5-CAT26-68	Contig13654	2,793	2,271	*GH48*	CBM 10	Y	6,953.9	Y	EXG	EXG (S, P)^e^	EXG (P)^f^

^a^*GH*: glycosyl hydrolase; ORF: open reading frame. ^b^The predicted signal peptide could be absent due to incomplete ORF sequence at the 5' end of contig sequence. ^c^Top score and e-value < 10^-10 ^as putative domain: endoglucanase (EG), exoglucanase (EXG), β-glucanase (β-glu), xylanase (Xyl) and chitinase. ^d^4-methylumbelliferyl-β-cellobiose (4-MUC) assays were used for broad cellulase activity analysis of endo-, exo-, β-glucanase and xylanase. S = supernatant; P = cell pellet. ^e^These cellulase constructs were chosen for subsequent enzymatic assays of dye CM-cellulose and *p*-nitrophenyl-β-D-glucopyranoside for their high 4-MUC activity expressed in *P. pastoris *(Figure 5). ^f^Low or undetectable levels of dye-CMC and *p*NPG activity. ^g^celD is a known gene from National Center for Biotechnology Information GenBank (Xue *et al*. [26]). The cloned sequences were submitted to GenBank (GenBank:JF906702-GenBank:JF906719).

Next, we investigated the expression of the major cellulases induced by rice straw according to RPKM value, which represented the normalized expression level from the transcriptome (see Additional file 2). Twenty-seven contigs showed a RPKM value > 500, which were the major components of induced cellulases, including the *GH1*, *GH3*, *GH4*, *GH6*, *GH10*, *GH11*, *GH38/GH57*, *GH43 *and *GH48 *families. Among these, contig2828 (*GH4*, malate dehydrogenase), contig13654 (*GH48*, cellulase Cel48A), contig3216 (*GH11*, xylanase), contig9299 (*GH6*, cellobiohydrolase II-like cellulase) and contig20167 (*GH6*, cellobiohydrolase II-like cellulase) are the top five genes of highest expression. *GH6 *(eight contigs) and *GH43 *(six contigs) are the two most highly represented families among these twenty-eight contigs. In short, the major cellulosic genes induced by rice straw are cellulases (BGLU, cellobiohydrolase, cellobiohydrolase II-like cellulase and cellulase Cel48A) and hemicellulases (xylanase, β-xylosidase and endo-1,4-β-xylanase).

To reveal the function of cellulase genes, the secretome mass data were subjected to BLAST analysis against the transcriptome EST data set. As shown in Table 4, 40 of the 220 unique *GH*-like contigs were also hit multiple times by these peptide sequences. Specifically, BGLU, endo-1,4-β-xylanase, xylanase B and Cel48A (*GH48 *family) constituted the major sequence annotations, among which the two xylanases of *N. patriciarum *were published [21,25]. The congruent results between the transcriptome and secretome analyses may imply that the enzymes mentioned above are the major protein constituents for degrading rice straw during processing. A good sequence database assembled from 454 data not only provides EST sequences for cDNA cloning but also helps to annotate the proteomic data more accurately than the previous online database (see Additional file 1). Taken together, both transcriptome and proteome analyses help one to explore the major functional enzymes involved in rice straw degradation. Such information may assist in the design of the CBP by combining the major cellulases in one microbe.

In addition to cellulases, our study also uncovered proteins associated with the cellulosome in rumen fungi, such as contig8798, which is annotated as a cellulosome enzyme dockerin type I protein, as well as many contigs that were predicted to contain the CBM10 domain (pfam02013). These conserved domains are responsible for assembly of multimeric cellulase/hemicellulase protein complexes and are the noncatalytic docking domains of cellulosomes found in anaerobic fungi [36,37]. Our results suggest that a possible cellulosome complex exists in *N. patriciarum *for the adhesion and hydrolysis of lignocelluloses.

However, we could not detect a large amount of secreted cellulase activity in the medium, because the cellulases of *N. patriciarum *W5 may form a complex cellulosome structure and bind to the surface of the cellulose substrates. The fact that many cellulases and BGLUs from *N. patriciarum *W5 have a dockering domain (CBM10) suggests that there are cellulosomes in *N. patriciarum *W5 on which cellulases might be located. The concentration of extracellular cellulase proteins of *N. patriciarum *W5 detected in our laboratory was about 138.2 to 193.7 mg/L, of which 30% showed xylanase activity.

### Validation and functional assay of potential novel cellulase genes

On the basis of transcriptome and secretome analyses, 19 highly expressed (high RPKM values) or potentially full-length contigs were selected for cellulase gene cloning. This serves two purposes: to validate the assembly predictions and to obtain actual sequences for functional expression.

These putative cellulase genes were then introduced into *Pichia pastoris *or *Saccharomyces cerevisiae *for expression. Enzymatic screening of these constructs by 4-MUC activity assay indicated that several putative cellulases function normally in these yeast hosts, including genes with homology to *GH *families *GH1*, *GH3*, *GH5*, *GH6*, *GH9 *and *GH48 *(Table 5). In addition, most of them carry the CBM10 domain, a cellulose binding module whose structure in solution has been resolved [38]. Significantly, some contigs assembled from the 454 data were almost identical to each other. Polymerase chain reaction (PCR) cloning using primer pairs from consensus regions among these contigs indeed led to identification of many respective sequences, suggesting the presence of transcript isoforms and/or gene families in the genome rather than sequencing or assembly errors. Taken together, these results show good sequence quality from the transcriptomic data and fast identification of desired genes by complementation of omics analyses.

**Figure 5:**
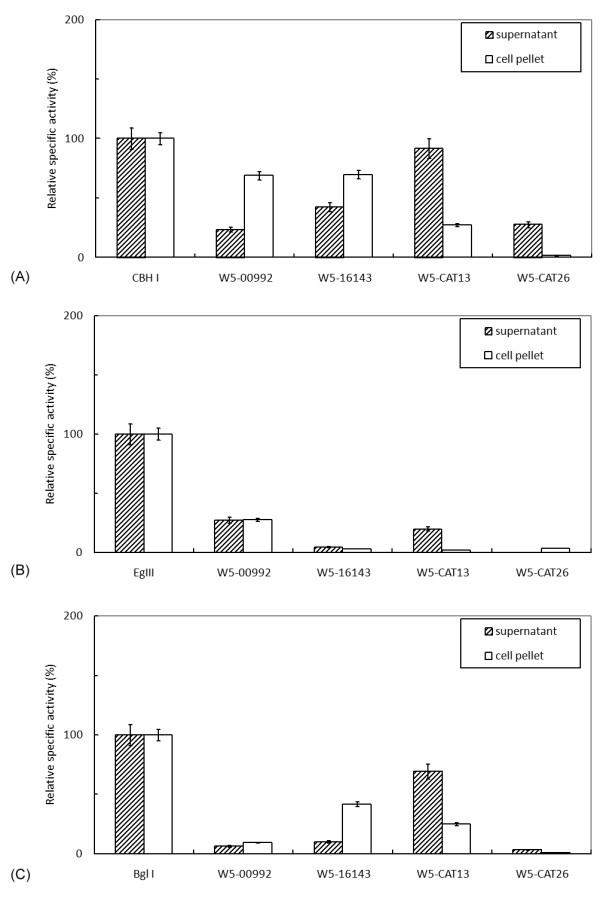
**Recombinant cellulase activities of W5 clones expressed in *Pichia pastoris***. **(A) **4-methylumbelliferyl-β-cellobiose (4-MUC assay) for exoglucanase activity and *Cbh*I as relative specific activity marker (100%). **(B) **Dye CM-cellulose (dye-CMC) assay for endoglucanase activity and *EgI*II as relative specific activity marker (100%). **(C) ***p*-nitrophenyl-β-D-glucopyranoside (*p*NPG) assay for β-glucosidase activity and *Bgl*I as relative specific activity marker (100%).

Four potential novel, functional cellulase genes were chosen for further *p*NPG and dye-CMC enzymatic assays (Table 5 and Figure 5). The cloned cellulase genes ranged between 1,989 and 2,361 bp long and had two or three predicted *N*-glycosylation sites. Two of them are putative BGLU genes, one of which belongs to the *GH3 *family (clone W5-16143) and the other of which belongs to the *GH1 *family (clone W5-CAT13). None of the BGLUs seems to have a cellulose binding domain. The exocellulase gene (clone W5-CAT26) belongs to the *GH48 *family and carries two CBMs at the C terminus. The endocellulase gene (clone W5-00992) belongs to the *GH9 *family and has three CBMs at the C terminus.

To screen for candidate clones and to determine the time window for optimal cellulase expression in *P. pastoris*, a time course of 4-MUC assays for culture supernatants was carried out at 24-hour periodicity for up to 5 days. We found that the maximum activities for all genes appeared 72 hours after induction. Although all cultures showed activities toward the 4-MUC substrate, the specific type of cellulase activity could be determined by the differential response to the other two substrates, dye-CMC and *p*NPG. As shown in Figure 5, the supernatant and cell pellet from each culture were used to assay the activities. All the results are presented as relative activities compared to the positive control as described in Methods. Their relative specific activities were determined by comparison to the benchmark enzymes, *Cbh*I and *EgI*II of *T. reesei *and *Bgl*I of *A. niger*, for EXG (4-MUC), EG (dye-CMC) and BGLU (*p*NPG), respectively.

In the assay for 4-MUC, the W5-CAT26 showed no activity in the cell pellets. In contrast, W5-00992 and W5-16143 showed very high activities (> 100%) in the pellets, suggesting that a large portion of the expressed cellulase was trapped inside the cell. In the dye-CMC assay, only the W5-00992 pellet showed apparent EG activity (27.5% relative to that of *EgI*II), while all other candidates displayed low activities in both supernatants and pellets (< 20% of *EgI*II's specific activity). In the *p*NPG assay for BGLU activity, W5-CAT13 showed appreciable activities (> 69% of *Bgl*I's specific activity) in the supernatant.

In summary, our results derived from the enzymatic assays and comparisons to the benchmarks are consistent with the protein functions predicted on the basis of their sequences. However, most of them showed a majority of the activities in the cell pellets, indicating that the native signal peptides of these cellulase genes could not be well recognized in *P. pastoris*. In the pursuit to find the best expression system, these genes were also introduced into *E. coli*, but most of the proteins appeared to be nonfunctional or showed low activity in this prokaryote (data not shown), possibly due to protein misfolding or improper posttranslational modifications. Parallel experiments using *S. cerevisiae *as the expression host were performed, but no activities were detected in the supernatants (Table 5), suggesting strong retention of these exogenous proteins inside the cells. Overall, *P. pastoris *was found to be a better host for expressing cellulase genes from W5, because the proteins were reasonably active and some of them were highly active in the supernatant. Future work is required to replace or modify the sequence of the signal peptide to enhance secretion and thus to increase extracellular activity.

Interestingly, a number of sequence variants were identified from cloning of W5-10151 using only one pair of PCR primers, among which at least four unique variants (W5-10151-39, W5-10151-38, W5-10151-5 and W5-10151-6) were found. To verify the sequence accuracy, the clones were checked by conducting a BLAST search against the 454 transcriptional contigs, and the cloning variants could be found in our contig data set (Table 5). Significantly, the one with the longest predicted ORF (W5-10151-39) displayed EXG activity when expressed in both yeast hosts.

## Conclusions

This study successfully verified 19 potential cellulase genes out of 219 putative *GH *family contigs using omics analyses, and 10 of them had detectable cellulase activity. By complementary application of the omics data, we identified four novel major cellulases from *N. patriciarum *W5 and expressed them in *P. pastoris *for functional expression. Characterizations of their cellulase activities imply that their enzymatic properties are competitive enough for potential commercialization.

Our transcriptomic data show high expression levels of the four cellulases in W5, and this was also supported by secretome analysis. In summary, this study showed that the secretome can be used to find major protein regions and that transcriptome analysis can predict full-length gene assembly and provide insights into feedstock metabolism. Thus, omics approaches can be combined to accelerate novel gene discovery and functional gene selection in a comprehensive manner, especially for organisms with little sequence information.

## Methods

### Microbial growth

The growth condition of *N. patriciarum *W5 was adapted from a rumen content of a water buffalo. The isolate was maintained in a rumen fluid-containing medium supplemented with 0.5% rice straw as the sole carbon and was anaerobically grown at 39°C for three or four days [19] with some variations. Glucose, carboxylmethyl cellulose (CMC), avicel or the powder of rice straw, napiergrass or sugarcane bagasse added to the broth (300 mL) as a sole carbon source to induce the cellulase production of *N. patriciarum *W5. To collect extracellular proteins from W5, four replicates of 300 mL of medium were cultured anaerobically in 39°C incubators for three to four days, and supernatants were harvested by centrifugation.

### Extracellular cellulase purification

A total of 1.5 L of culture supernatants were filtered through a 0.2-mm membrane (Sartorius, Goettingen, Germany), then dialyzed and condensed 100-fold using Viva Flow 50 (10-kDa cutoff) (Sartorius) against starting buffer (50 mM Tris·HCl, pH 7.0) to a final volume of 20 mL at 4°C.

To purify the protein fractions that contain cellulases, sequential FPLC (ÄKTAFPLC; GE Healthcare, Taipei, Taiwan) was conducted. First, the samples were subjected to a HiPrep 16/10 QFF anion exchange column (GE Healthcare, Taipei, Taiwan) preequilibrated with 60 mL of starting buffer to stabilize the UV signal, and then a 10-mL sample was applied and eluted for 60 mL with a step gradient using a 15% mixing ratio of elution buffer (50 mM Tris·HCl, 2 M NaCl, pH 7.0) at a constant flow of 3 mL/minute. Fractions of 5 mL from the eluates were collected and assayed for the activity of *p*NPG, dye-CMC and 4-MUC (Sigma-Aldrich, St Louis, MO, USA), respectively. Fractions with apparent activity were pooled, condensed and dialyzed against gel filtration buffer (20 mM Tris·HCl, 150 mM NaCl, pH 7.0) with Viva Spin (10-kDa cutoff). Four milliliters of the condensed sample were applied to the HiLoad 16/60 Superdex 200 gel filtration column (GE Healthcare, Taipei, Taiwan) with a constant flow of 1.2 mL/minute. All 3-mL fractions were assayed for enzyme activity using *p*NPG, dye-CMC and 4-MUC as substrates. Fractions displaying the activity of BGLUs with EXGs (*p*NPG and 4-MUC) and EGs (dye-CMC) were pooled and condensed, respectively.

### Enzyme activity assay

4-MUC assays were carried out for broad quantitative analysis of cellulase activity, and the activity was measured by fluorometry with excitation and emission wavelengths at 365 and 450 nm, respectively. Dye-CMC was used as a substrate for the quantification of EG activity, and the activity was measured at 590 nm. *p*NPG was used as the substrate for assays of BGLU activity, and the activity was measured at 410 nm. If both dye-CMC and *p*NPG activity was low, the 4-MUC activity represented the exocellulase activity. Protein concentration was measured with the Protein Assay reagent (500-0006; Bio-Rad Laboratories, Hercules, CA, USA). The specific activities were defined as digesting 1 μM substrate per milligram of protein per hour.

### SDS-PAGE

SDS-PAGE was performed to examine the approximate protein sizes [39]. Proteins were separated on 12% polyacrylamide gels and stained with Coomassie Brilliant Blue R-250 (Bio-Rad Laboratories). The molecular weight markers were obtained from Fermentas (Burlington, ON, Canada).

### Zymogram assay

A total of 100 μg of pooled fractionated P2-1 and P2-2 were separated with 17 × 17 cm of 12% SDS-PAGE controlled by the PROTEAN II xi electrophoresis system (Bio-Rad Laboratories). Duplicated samples were separated on the same 12% SDS-PAGE gel using non-β-ME sampling buffer and premixed without heating for zymogram tests. SDS-PAGE for the zymogram test was laid onto a 16-cm diameter of 1% CMC (for EG; Calbiochem, Darmstadt, Germany); catalog no. 217277; Merck & Co., Whitehouse Station, NJ, USA) agar plate prespread with 500 μL of 2% 4-MUC (for EXG), air-dried and finally moisturized with 0.5 mL of 90 mM sodium acetate on top of the PAGE gel and kept at 30°C for three hours or overnight to detect the EXG or EG activity, respectively. The 4-MUC plate was measured by fluorometry under UV light at 365 nm for qualitative activity. The CMC plate was later stained with 15 mL of Congo Red (1 mg/mL) at room temperature for 40 minutes and then destained with 1 M NaCl to visualize reactive (transparent) bands. The transparent bands on the zymogram were estimated for size guided by molecular weight markers, and the corresponding bands on SDS-PAGE were sliced from the same size range, dissolved and digested with trypsin solution, and subjected to ESI-TRAP LC-MS/MS analysis.

### In-gel trypsin digestion

The protocol used for in-gel trypsin digestion of proteins in gels was adapted mainly from a method described previously [40]. Briefly, the protein bands from one-dimensional gel were excised, and each band was diced into small pieces (about 0.5 mm^3^). The gel pieces were placed into microfuge tubes and washed a few times with solution containing 50% methanol and 5% acetic acid for two to three hours, followed by two washes with a solution of 25 mM NH_4_HCO_3 _in 50% acetonitril for 10 minutes each, and then the gel pieces were dried in a vacuum centrifuge. Reduction with dithiothreitol (DTT) and alkylation with iodoacetamide of proteins in gel pieces were performed, and the gel pieces were washed and dried in a vacuum centrifuge before trypsin digestion. A 25- to 40-μL trypsin solution in 25 mM NH_4_HCO_3 _containing 75 to 100 ng of sequencing grade modified trypsin (Promega, Madison, WI, USA) was added and incubated with gel pieces for 12 to 16 hours at 37°C. To recover the tryptic peptides, a solution of 30 μL containing 5% formic acid and 50% acetonitril was added to the gel pieces, agitated in a vortex for 30 to 60 minutes and transferred into a new tube. The remains were repeated once with 15 μL of solution, and the resulting two eluates were combined and dried in a vacuum centrifuge. The dried pellet was redissolved in 10 to 20 μL of 0.1% formic acid for LC-MS/MS analysis (Proteomics Core Laboratory, IPMB, Academia Sinica, Taipei, Taiwan).

### MS method for protein identification and analysis

An LTQ mass spectrometer model coupled with an online capillary LC system from Thermo Fisher Scientific (Waltham, MA, USA) was utilized for protein identification and analysis. The capillary LC system equipped with an autosampler and Surveyor pumps (Thermo Fisher Scientific), a C18 trap column (100 μm × 3.0 cm, 5 μm, 200-Å Magic C18 AQ resin; Michrom Bioresources, Inc., Auburn, CA) and a C18 reverse phase column (75 μm × 11 cm, 5 μm, 100-Å Magic C18 resin, Auburn, CA) was used to deliver solvent and to separate tryptic peptides with a linear gradient from 5% to 40% of acetonitril in 0.1% (vol/vol) formic acid for 40 minutes (or 60 minutes) at the nanoflow rate of approximately 300 nL/minute. A sample volume of 5 μL was loaded onto the column. The C18 reverse phase column was coupled to a nanoelectrospray ionization source, and the acquisition of the data was conducted with a full MS scan followed by four MS/MS scans of the top four precursor ions from the MS scan. The MS scan was conducted over the mass-to-charge (*m*/*z*) range 400 to 1,800 using the data-dependent data acquisition mode with dynamic exclusion enabled. The acquired ESI-TRAP MS/MS data were analyzed using a MASCOT MS/MS Ion Search program (http://www.matrixscience.com/) against the ORFs of our transcriptome database. The peak list data (DTA) used for the database search were generated from MS/MS spectra using an ion intensity threshold of 1,000 and a minimum ion count of 10. The protein sequences in the database were indexed with trypsin digestion with two missed cleavages, the 600 to 3,500 molecular weight range, a variable modification of Met by oxidation and a static modification of Cys by carboxyamidomethylation. The DTA data were searched with 1.0 atomic mass units (u) of precursor peptide mass tolerance and 0.5 u of fragment mass tolerance. The matched peptides were accepted when they passed multiple filters, *X*_corr _≥ 1.5 for singly charged ions (*z *= 1), *X*_corr _≥ 2.0 for doubly charged ions (*z *= 2), *X*_corr _≥ 2.5 for triply charged ions (*z *= 3), ΔCN ≥ 0.1 and peptide probability ≤1 × 10^-3^. Matched proteins were accepted only when they had at least two distinct peptide hits.

### RNA extraction and cDNA synthesis of W5

The cells were cultured under anaerobic conditions at 39°C for 2.5 days, and then the medium was removed by filtration to collect the cells. The culture mass was ground in liquid nitrogen, and total RNA was extracted using the hot acid phenol-chloroform method and quantified on a spectrophotometer. From each substrate-induced culture (CMC, avicel, cellobiose, rice straw, sugarcane and napiergrass), equal amounts of total RNA were pooled together. The mRNA of the pooled cellulose-induced total RNA samples were enriched using the Dynabeads mRNA Purification Kit (Invitrogen, Carlsbad, CA, USA), and double-stranded cDNA was synthesized from the 10 μg of purified mRNA sample using the SuperScript Double-Stranded cDNA Synthesis Kit (Invitrogen) according to the manufacturer's instructions. The cDNA were then fragmented and prepared for the shotgun libraries according to Roche's protocols. The deep sequencing experiments were carried out on the GS FLX Sequencer by Mission Biotech Co., Ltd. (Taipei, Taiwan).

To synthesize the rice straw-induced cellulase containing cDNA for reverse transcriptase (RT)-PCR and gene cloning, reverse transcription using total RNA was carried out with the SuperScript™ II Reverse Transcriptase Kit (Invitrogen). The reaction contained the total RNA (4 μg), oligo(dT) primer, BD SMART II A oligo (Clontech, Mountain View, CA, USA), 4 μL of 5 × RT buffer, 10 mM DTT, 0.5 mM each of deoxyribonucleotide triphosphate and 10 U of SUPERSCRIPT™ II RNase H Reverse Transcriptase (Invitrogen). The single-stranded DNA product was stored at -20°C.

### Transcriptome analysis: cDNA assembly, ORF prediction and domain prediction

The 454 sequence reads were processed through SeqClean to remove poly(T) stretches and then assembled into unique contigs by CAP3 (read minimum overlap length = 40 with minimum identity = 0.9). ORFs were predicted by GETORF, a part of EMBOSS software [32], for in-frame regions between a start and a stop codon. All predicted ORFs were then submitted to local RPS-BLAST [33] and the NCBI CDD database to retrieve domain predictions with an e-value < 10^-5^.

For quantitative profiling, an RNA-Seq library was constructed from the cDNA (synthesized from rice straw-induced RNA) using the Illumina kit, and paired-end sequencing was performed on the Illumina GA2 sequencer of the High Throughput Sequencing Core at Academia Sinica, Taipei, Taiwan. Reads from PE2*40 nucleotides (7.4 million pairs) (insert range between 180 and 220 bp) were obtained. The data were mapped to the EST contigs derived from 454 assembly and PCR cloning, and the relative expression levels were calculated by the RPKM method [35] using CLC-bio Genomics Workbench software (CLC bio, Aarhus N, Denmark) and sorted by ranking. The contigs with ORFs longer than 500 bp were further analyzed for *GH *family gene classification. The sequences with the highest RPKM scores and significant e-values (< 10^-10^) were selected for functional annotation. The analysis flowchart is illustrated in Figure 4.

### Strains and plasmids used for cloning and expression

The *E. coli *DH5α strain was used as a host for cloning and plasmid propagation. The strain was cultured in a low-salt LB medium. *P. pastoris *GS115 strain (Invitrogen) was used as a host for expressing cloned cellulase genes. The *P. pastoris *transformants were selected in YPD (1% yeast extract, 2% peptone, 2% dextrose) plates containing 100 μg/ml of zeocin. For expression, the BMGY medium and the BMMY medium were used and prepared according to the *Pichia *expression system manual (Invitrogen). The *S. cerevisiae *transformants were selected in SC-URA plates. The pGEM-T easy vector (Promega) was used for gene cloning and sequencing. pPICZ A plasmid (Invitrogen) and pRS426 plasmid with glyceraldehyde 3-phosphate dehydrogenase (GAPDH) promoter were used for protein expression in *P. pastoris *and *S. cerevisiae*, respectively.

### Cloning and construction for selected cellulase genes

The reverse-transcribed W5 cDNA was used to clone the selected cellulase genes by PCR using the gene-specific oligonucleotide primers the sequences of which were derived from the transcriptome contigs (Table 5). For each pair, the forward primer was engineered with *Xho*I (New England Biolabs, Ipswich, Massachusetts, USA) and the reverse primer was engineered with *Eco*RI (New England Biolabs) recognition sequences to allow for directional cloning into expression vector. After amplification, each PCR was cloned into the pGEM-T easy vector for sequencing. Plasmid DNA from the positive clones were digested by *Xho*I and *Eco*RI, and the putative cellulase gene was subcloned into the pPICZ A vector, which contained an AOX1 promoter that can be induced by methanol, and allowed for selection by the antibiotic zeocin. For expression in *S. cerevisiae*, the ORF of the candidate cellulase gene was fused with the GAPDH promoter and cloned into the pRS426 plasmid and then transformed into yeast cells. The procedures for plasmid preparation, restriction digestion, ligation and *E. coli *transformation were carried out in accordance with the manufacturers' protocols.

### Transformation and cellulase expression in *Pichia pastoris*

For growing *P. pastoris *GS115 cells, all cultures were incubated at 30°C with 250-rpm orbital shaking. Procedures for preparation of competent cells were carried out according to the manufacturer's description (Bio-Rad Laboratories). For transformation, 10 μg of each cellulase construct in the pPICZ A vector was linearized with *Sac*I (New England Biolabs) and transformed into yeast *P. pastoris *GS115 strain by the Gene Pulser Xcell (Bio-Rad Laboratories). The 200-μL electroporated mixtures were plated into YPD medium containing 100 μg/mL zeocin and incubated at 30°C until colonies appeared. The introduction of each cellulase gene into the yeast cell was further confirmed by PCR using the forward and reverse primers of AOX1 promoter (Invitrogen). For expression and induction of the cellulase genes, the protocols were performed according to the *Pichia *expression system manual (Invitrogen). To monitor the activities of expressed proteins, 1-mL samples from culture were collected every 24 hours. After 1-mL samples were taken, methanol (100%) was added to a final concentration of 0.5% (vol/vol) to maintain induction. To screen for the *Pichia *transformants expressing high activity of cellulase protein, ten colonies were randomly selected from YPD/zeocin plates and individually cultured in 10 mL of BMMY at 30°C for five days for methanol induction.

### Transformation and cellulase expression in *Saccharomyces cerevisiae*

Yeast BY4741 (MATa his3Δ1 leu2Δ0 met15Δ0 ura3Δ0), a descendant of S288C, was grown overnight at 30°C with 250-rpm shaking. Procedures for the preparation of competent cells were carried out according to the manufacturer's description (Bio-Rad Laboratories). For transformation, 200 ng of each cellulase construct in pRS426 was directly transformed into yeast BY4741 strain using the Gene Pulser Xcell Electroporation System (Bio-Rad Laboratories). The 50-μL electroporated mixtures were incubated in 1 mL of YPAD (1% yeast extract, 2% peptone, 0.04% adenine sulfate, 2% dextrose) and recovered after three hours of incubation at 30°C with 250-rpm shaking. The transformants, after being centrifuged and washed twice with 1 mL of deionized distilled water, were selected on the SC-URA plate at 30°C until URA^+ ^colonies appeared. Only the strains that carried the desired plasmids will survive and form colonies on the SC-URA plate. Ten colonies of transformants from each strain were randomly selected and cultured in 5 mL of SC-URA medium at 30°C for 24 hours, and the harvested supernatants were then screened by the 4-MUC activity. At least three higher-expression colonies of each gene were diluted to 600-nm optical density (OD_600_) = 0.1 and grown in 50 mL of SC-URA medium at 30°C for 24 hours. The condensed supernatants and collected cell pellets were assayed again.

### Functional screening and activity assay

For the 4-MUC enzymatic assay, 40 μL of the supernatants from the 1-mL sample (collected as described above) were added to reaction buffer (90 mM NaOAc, pH 5) containing 2 mg/mL 4-MUC (Sigma-Aldrich) and incubated for 20 hours at 30°C before addition of stop solution (2% Na_2_CO_3_, pH 11). The activity was determined by measuring absorbance at OD_450 _nm (excitation at 365 nm) using the SpectraMax M2 Microplate Reader (Molecular Devices, Sunnyvale, CA, USA).

To find the best candidates from the ten transformants, 4-MUC assays were repeated to select three colonies that displayed the highest activity for each specific gene. The culture condition was the same as above, except for scaling up culture to 50 mL of BMMY.

To evaluate the activities of the cell pellet, the protein extraction procedure was based on the *Pichia *expression system manual (Invitrogen). For the 4-MUC enzymatic assay, 40 μL of the pellet extractions from the 1-mL culture harvest were added to reaction buffer (90 mM NaOAc, pH 5) containing 4-MUC at 2 mg/mL and incubated for 2.5 hours at 30°C before addition of stop solution (2% Na_2_CO_3_, pH 11). The activity was determined as described above.

For determination of the activities in benchmarks, the genes of *Cbh*I (an exoglucanase of *T. reesei*), *EgI*II (an endoglucanse of *T. reesei*) and *Bgl*I (a β-glucosidase of *A. niger*) were also cloned into the pPICZ A vector and transformed in the *Pichia *expression system. The primers of these genes were derived from the sequences at the NCBI. Cloning and construction were carried out the same way as for W5 genes. The calculation of specific activity in each cellulase candidate was derived from the raw-read/protein concentration. Then the activities of these cellulases were used as controls to derive relatively specific activity of W5 enzymes for exoglucanse (4-MUC), endoglucanase (dye-CMC) and β-glucosidase (*p*NPG), respectively.

## Abbreviations

4-MUC: 4-methylumbelliferyl-β-cellobiose; BGLU: β-glucosidase; CAZy: Carbohydrate-Active enZYmes database; CBP: consolidated bioprocess; CMC: carboxylmethyl cellulose; dye-CMC: dye CM-cellulose; EG: endoglucanase; EXG: exoglucanase; *GH*: glycosyl hydrolase; LC: liquid chromatography; *p*NPG: *p*-nitrophenyl-β-D-glucopyranoside; RPKM: Reads Per Kilobase of exon model per Million mapped reads; RT-PCR: reverse transcriptase-polymerase chain reaction.

## Competing interests

The authors declare that they have no competing interests.

## Authors' contributions

TYW, HLC, MYJL, YCC and HMS designed, coordinated and performed some of the experiments and/or analyses and also wrote the manuscript. WHL and MCH supervised the research and helped to draft and revise the manuscript. CTM, SKR, YCW, HYC HKM, KYH and YWT performed the culturing, cloning and functional experiments. CKC, YHC, JYL and SPC performed next-generation sequencing experiments. TYH, TCC and ACCS performed bioinformatics analyses. All authors read and approved the final manuscript.

## Supplementary Material

Additional file 1 **MASCOT results of BLAST homology search of 24 cellulase-like proteins in fungal species in the SwissProt database**. Click here for file

Additional file 2 **Categorization of the 219 glycosyl hydrolase-like contigs and their expression levels by Reads Per Kilobase of exon model per million mapped reads (RPKM) value**. Click here for file
