# Supplementary material for: Functional characterization of cellulases identified from the cow rumen fungus Neocallimastix patriciarum W5 by transcriptomic and secretomic analyses
Source: Biotechnol Biofuels. 2011 Aug 17;4:24. doi: 10.1186/1754-6834-4-24 (PMC3177772; doi:10.1186/1754-6834-4-24)
Supplement: Additional file 1 — MASCOT results of BLAST homology search of 24 cellulase-like proteins in fungal species in the SwissProt database. [file 1754-6834-4-24-S1.DOC]

**Additional file 1. MASCOT results of BLAST homology search of 24 cellulase-like proteins in fungal species of SwissProt database.**

| **Accession #** | **Annotation [species]** | **Peptide** |
| --- | --- | --- |
| **CAB92325** | Endoglucanase 45A [*Piromyces equi*] | K.KGSYGAVSLK.S |
|  |  | K.DAGSNTKAK.Y |
| **AAD43818/AF165266** | Endoglucanase precursor [*Piromyces rhizinflata*] | K.LGYKCCK.S |
| **AAP30753** | Cellulosomal glycoside hydrolase family 9 endoglucanase Cel9B [*Piromyces* sp. E2] | K.EPKPDVK.W |
| **Q12647** | GUNB_NEOPA Endoglucanase B precursor (Endo-1,4-beta-glucanase B) (Cellulase B) | K.QIATYFK.D |
|  |  | K.QVVDDLR.Y |
|  |  | R.FVIQGIPVIITK.Y |
|  |  | R.QAFNLKIR.G |
|  |  | K.FGTNESTMANQK.F |
|  |  | K.CLNIPK.N |
| **ABY52798** | 1,4-beta-D-glucan-cellobiohydrolase [*Piromyces rhizinflatus*] | R.SGLSFDSTK.T |
|  |  | K.EEGLVVER.N |
|  |  | K.ASLVGHSDDNASEDTGK.L |
| **AAD51054/AF174361** | Exocellobiohydrolase Cbh120 [*Piromyces rhizinflata*] | R.LVELK.I |
| **AAL92497** | Exoglucanase Cel6A [*Piromyces sp.* E2] | K.LGYKCCK.G |
|  |  | K.MV |
| **AAM94167** | Cellulosomal glycoside hydrolase family 6 exoglucanase Cel6A [*Piromyces equi*] | R.TIRVAK.L |
| **AAN76734/AF449412** | Cellulase Cel48A precursor [*Piromyces sp.* E2] | K.ATDYDYIGLDYAATYK.Q |
|  |  | K.LDVYTTQR.G |
|  |  | K.LGDYLR.Y |
|  |  | K.QYFSVR.Q |
|  |  | K.VPNFIDLAFQTAR.K |
|  |  | R.YGPSIDGDTNFK.Q |
|  |  | K.FTVGNGQNQHK.G |
|  |  | K.NLDVEK.G |
|  |  | K.VCQEWAK.W |
|  |  | R.VNGGEVVHATNLEWSGNPDEWNASNFNK.E |
|  |  | K.DWATSLER.Q |
|  |  | K.FNEDVYIPSGWTGK.N |
|  |  | K.IGNCVGYQQCQAGSGK.N |
|  |  | K.LTGDYSGVEK.A |
|  |  | K.LTGDYSGVEK.A |
|  |  | K.WGGQNGFLDLFTLDNSYSK.Q |
|  |  | K.YKEDPDWAQVENFLNGGEAPR.F |
|  |  | R.QIELFR.W |
|  |  | R.VSGGEIVHATNLSWDGSPDEWNSSNFNK.A |
|  |  | R.YNPSSPATYAPEDDDITNYPAR.L |
|  |  | R.YTAAPDADAR.A |
|  |  | K.IFQMDHTGPTINGGSETFK.Q |
|  |  | K.MVTIDGAQYK.I |
|  |  | R.GNFLAR.R |
|  |  | R.LCVYGWFQNR.G |
| **AAN76735/AF449413** | Cellulase Cel48A precursor [*Piromyces equi*] | K.LGDYLR.Y |
|  |  | R.SGLSFDSTK.T |
|  |  | R.YGPSIDGDTNFK.Q |
|  |  | K.IGNCIGYQK.C |
|  |  | K.VCQEWAK.W |
|  |  | R.QLELFR.W |
|  |  | R.YTAAPDADAR.A |
| **AAM81966/AF459452** | Cellulase Cel9A precursor [*Piromyces sp.* E2] | K.KAETPDITVK.A |
|  |  | R.ILNLNLR.F |
|  |  | K.LGYKCCK.G |
|  |  | K.AAKPDFNIYTLWGALAGGPDK.S |
|  |  | K.EGSAWGSEEDGTK.C |
|  |  | K.EIYDFADQYR.G |
|  |  | K.MIIGVELFLVLLKK.N |
|  |  | K.NAGQWKYILDAVR.W |
|  |  | K.SYQLTTSYNK.Y |
|  |  | R.ATGDESYLDK.Y |
|  |  | R.FAFQNYDNNK.D |
|  |  | K.AETPDIR.L |
| **CAC34952** | Beta-glucosidase [*Piromyces sp.* E2] | R.YKGLIK.N |
|  |  | K.LPSDFK.W |
|  |  | K.QYLENLR.L |
|  |  | R.VGLNWFSK.R |
|  |  | K.YGPTIR.V |
|  |  | K.YANLAAEFR.I |
|  |  | K.RYEGLIK.N |
| **AAO41704** | Beta-glucosidase precursor [*Piromyces sp.* E2] | K.IAVIGK.D |
|  |  | K.VLLEAGESK.E |
|  |  | -.LDDAVLR.I |
|  |  | K.NDESVLPIK.G |
|  |  | R.IDSFGAGLDMNMPGGK.Y |
|  |  | R.KVGEAQGNEFYQR.G |
|  |  | R.LDDAVLR.I |
|  |  | R.LDDAVLR.I |
|  |  | R.LNLDLWHGGNELIEAVAAVNK.N |
|  |  | K.ADYEAVLAALK.D |
|  |  | K.LNPVIMADGPAGLR.L |
|  |  | K.MTVYDYNEGLFVGYR.W |
|  |  | K.VGADSNVLLK.N |
|  |  | R.EWCADLTNEEK.I |
|  |  | K.AREWCADLTNEEK.I |
|  |  | K.ELTTDILR.T |
|  |  | K.GTATSWQAGINNAATFDR.Q |
|  |  | K.VAADSTVLLK.N |
|  |  | R.EDFPTDVNYDPSLPGGGEEK.T |
|  |  | R.FAKGTATSWQAGINNAATFDR.Q |
|  |  | R.GINFALGPAMNIQR.A |
|  |  | R.IIRTLYK.A |
|  |  | R.EDYPTDVK.Y |
|  |  | K.EKLISR.F |
|  |  | K.DSMPAK. |
|  |  | K.DSMPAK.F |
|  |  | K.ISLVTGR.E |
|  |  | K.FVKPEEGEYTVFAGSNAR.D |
|  |  | K.GIQSQGVIATSK.H |
|  |  | K.YVIDPLSAITER.A |
|  |  | R.LNLDLWHGGNELIDAVASVNK.N |
|  |  | K.VGEAQGNEFYQR.G |
|  |  | R.LDDAALR.I |
|  |  | R.LDDAALR.V |
|  |  | K.IAIIGK.D |
|  |  | K.YEPEYPDGGEK.M |
|  |  | K.GLCLQDGPAGVR.F |
|  |  | K.NTIVVIHAPGPVNVPFLDK.V |
|  |  | K.ISIITGR.E |
|  |  | K.GMQSSGVIATSK.H |
|  |  | K.DSMPANFCEDMR.C |
|  |  | R.LLTDILK.D |
|  |  | R.LDDAVR.R |
| **AAP30744** | Beta-glucosidase Cel1B [*Piromyces sp.* E2] | K.NGPGKAPYR.C |
| **AAP30745** | Beta-glucosidase Cel1C [*Piromyces sp.* E2] | K.NEDGTYVEPPSDEEASYDNSKR.D |
|  |  | K.IMNANHYR.F |
|  |  | R.YKGLIK.N |
|  |  | K.YANLAAEFR. |
|  |  | K.GRGDGWTCIPPTLGSQAGSSWNTK.Y |
|  |  | K.WGAATAAYQVEGAWNEDGR.G |
|  |  | R.GDGWTCIPPTLGSQAGSSWNTK.Y |
|  |  | K.QYLENLR.L |
|  |  | K.YGGWLDYR.S |
|  |  | R.VGLNWFSK.R |
|  |  | K.NGPGKAPYR.C |
|  |  | R.LGPGKAPYR.C |
|  |  | R.GESVWDHFTHLYPK.N |
|  |  | K.YGPTIR.V |
|  |  | K.YANLAAEFR.I |
|  |  | K.DLPEFTAEEK.K |
|  |  | K.RYEGIIK.N |
|  |  | K.RYEGLIK.N |
|  |  | R.LAYVEDGVDVR.G |
|  |  | K.NWITINEPWVNCVSGYR.L |
|  |  | R.FSISWSR.L |
|  |  | K.FDEDIK.M |
|  |  | K.GWITINEPWVNCVAGYK.N |
|  |  | K.LPSDFK.W |
|  |  | K.LEDVWCSHNILLGHAQAVK.V |
|  |  | R.LEDVWCSHNILLGHAKAVK.V |
|  |  | K.DSEELPANRPPQK.L |
|  |  | R.IGWYSDPVMFGDYPESVK.Q |
| **AAL01214/AF177207** | Endo-glucanase CelJ [*Orpinomyces sp.* PC-2] | K.DGEFTIATGSGMR.C |
|  |  | K.RPETPDIR.L |
|  |  | K.VTQASDCTPYK.I |
| **AAC09228** | Cellobiohydrolase II [*Orpinomyces sp.* PC-2] | K.VNNVIVQNLK.F |
| **AAL01211/AF177204** | Cellobiohydrolase II-like cellulase CelH [*Orpinomyces sp.* PC-2] | K.KLGVFANVALY.- |
| **AAL01212/AF177205** | Cellobiohydrolase II-like cellulase CelI [*Orpinomyces sp.* PC-2] | K.IPITVPK.N |
|  |  | K.SIITPQISGLR.N |
|  |  | R.VNSDALDLFK.E |
|  |  | R.VNSDALDLFKEQLGF.- |
|  |  | K.YGIESGTGDLSRPTLINQENPYLPFFTTK.E |
|  |  | R.NADAIGVLKK.Y |
|  |  | R.LGYKCCK.K |
|  |  | R.NNIEMAQR.L |
|  |  | R.EILLR.N |
| **AAB69347** | Cellulase [*Orpinomyces joyonii*] | K.SLDLNR.W |
|  |  | -.VIAGSNAMR.N |
| **AAC05164** | 1,4-beta-D-glucan-4-glucanohydrolase [*Orpinomyces sp.* PC-2] | K.FGTNESTMANQK.- |
|  |  | K.AAASALSVAEVK.A |
|  |  | K.NLESAK.K |
| **AAD45834/AF016864** | Beta-glucosidase [*Orpinomyces sp.* PC-2] | R.GESVWDHFTHEYPK.N |
|  |  | K.NGIVITENGCAQPNYKVAR.A |
|  |  | K.NVESGDRTIDYSTNGNR.A |
|  |  | K.FAPTIR.V |
|  |  | K.GRGDGWTCIPPTLGSQAGSSWNTK.F |
|  |  | K.WGAATAAYQVEGAWDEDGR.G |
|  |  | K.WGAATAAYQVEGAWDEGNR.G |
|  |  | K.YFESIGQPQYADTYGEEDIEDESNINGTILHDK.Y |
|  |  | R.GDGWTCIPPTLGSQAGSSWNTK.F |
|  |  | K.NEDGSYVEPPTAEEANFDNSKK.D |
|  |  | R.LGPGKAPYR.C |
|  |  | K.NGIVITENGCAQPNYK.V |
|  |  | R.FGMTYIDFYK.D |
|  |  | K.DSLEHLGQWYLENVEQN.- |
|  |  | K.YANLAAEFR.I |
|  |  | R.FGMTYIDFYKDENLTR.H |
|  |  | R.LAYAVDGIDVR.G |
|  |  | R.VGLNWFNDR.Y |
|  |  | K.YRQDWYDQYLK.N |
|  |  | R.TIDYSTNGNR.A |
|  |  | R.IGWYSDPPMTGDYPASVK.E |
|  |  | K.GSSDFLGWNTYTAHWAAQVK.N |
|  |  | K.NWITINEPWVNCVSGYR.L |
|  |  | R.FSISWSR.I |
|  |  | K.LPSDFK.W |
|  |  | K.LEDVWCSHNILLGHAQAVK.V |
|  |  | R.LEDVWCSHNILLGHAKAVK.V |
|  |  | K.NEDGSYVEPPTAEEANFDNSK.K |
| **AAQ09256** | Cellobiohydrolase C precursor [*Neocallimastix sp.* W-1] | K.NNNKNNNNK.N |
| **ABY52793** | Cellobiohydrolase [*Neocallimastix patriciarum*] | R.IIINR.T |
