# Supplementary material for: Functional characterization of cellulases identified from the cow rumen fungus Neocallimastix patriciarum W5 by transcriptomic and secretomic analyses
Source: Biotechnol Biofuels. 2011 Aug 17;4:24. doi: 10.1186/1754-6834-4-24 (PMC3177772; doi:10.1186/1754-6834-4-24)
Supplement: Additional file 2 — Categorization of the 219 glycosyl hydrolase-like contigs and their expression levels by Reads Per Kilobase of exon model per million mapped reads (RPKM) value. [file 1754-6834-4-24-S2.DOC]

**Additional file 2. Categorization of the 219** GH-like contigs and their expression levels by RPKM value.

| **GH family** | **RPKM** | **contig name** |
| --- | --- | --- |
| GH1 | NA | Contig13432 |
|  | NA | Contig4463 |
|  | 2,080.7 | Contig8146 |
|  | 1,043.9 | Contig16557 |
|  | 532.1 | Contig1907 |
|  | 124.2 | Contig19120 |
|  | 0.3 | Contig7663 |
| GH3 | NA | Contig9462 |
|  | 374.8 | Contig8008 |
|  | 335.1 | Contig17778 |
|  | 141.4 | Contig4878 |
|  | 63.6 | Contig8929 |
|  | 38.6 | Contig16757 |
|  | 32.3 | Contig8087 |
|  | 26.1 | Contig11849 |
|  | 24.7 | Contig4604 |
|  | 18.6 | Contig14924 |
| GH4 | 9,043.7 | Contig2828 |
|  | 584.5 | Contig7536 |
|  | 85.6 | Contig18449 |
| GH5 | NA | Contig1072 |
|  | NA | Contig1200 |
|  | NA | Contig13796 |
|  | NA | Contig14061 |
|  | NA | Contig14560 |
|  | NA | Contig15085 |
|  | NA | Contig1618 |
|  | NA | Contig309 |
|  | 470.1 | Contig9346 |
|  | 311.2 | Contig12293 |
|  | 286.1 | Contig13743 |
|  | 211.3 | Contig12690 |
|  | 195.0 | Contig16942 |
|  | 140.0 | Contig13579 |
|  | 81.2 | Contig10182 |
|  | 69.3 | Contig3314 |
|  | 36.9 | Contig3879 |
|  | 36.0 | Contig10206 |
|  | 20.2 | Contig7622 |
|  | 10.7 | Contig16883 |
| GH6 | NA | Contig1380 |
|  | NA | Contig14575 |
|  | NA | Contig1458 |
|  | NA | Contig19950 |
|  | NA | Contig2206 |
|  | NA | Contig4691 |
|  | NA | Contig8664 |
|  | NA | Contig9776 |
|  | 3,867.8 | Contig9299 |
|  | 3,166.6 | Contig20147 |
|  | 2,498.3 | Contig15588 |
|  | 2,479.6 | Contig8755 |
|  | 1,335.7 | Contig19058 |
|  | 907.9 | Contig16396 |
|  | 693.6 | Contig18112 |
|  | 599.4 | Contig10151 |
|  | 377.2 | Contig9839 |
|  | 318.1 | Contig7605 |
|  | 214.9 | Contig13874 |
|  | 206.9 | Contig12553 |
|  | 193.2 | Contig10478 |
|  | 177.4 | Contig8363 |
|  | 174.6 | Contig2687 |
|  | 121.0 | Contig16236 |
|  | 78.7 | Contig4514 |
|  | 77.1 | Contig6730 |
|  | 33.5 | Contig19865 |
|  | 31.1 | Contig8191 |
|  | 30.4 | Contig8337 |
|  | 26.0 | Contig8603 |
|  | 25.7 | Contig250 |
|  | 24.5 | Contig5997 |
|  | 22.1 | Contig6374 |
| GH8 | 128.1 | Contig16622 |
|  | 79.5 | Contig11888 |
| GH9 | NA | Contig6129 |
|  | 312.5 | Contig15560 |
|  | 285.9 | Contig6878 |
|  | 209.4 | Contig12773 |
|  | 149.4 | Contig10733 |
|  | 139.4 | Contig8960 |
|  | 107.0 | Contig17862 |
|  | 97.0 | Contig1361 |
|  | 91.2 | Contig4786 |
|  | 63.1 | Contig8764 |
|  | 25.4 | Contig9556 |
|  | 14.9 | Contig5557 |
| GH10 | NA | Contig10513 |
|  | NA | Contig19748 |
|  | NA | Contig20157 |
|  | NA | Contig2196 |
|  | NA | Contig3792 |
|  | NA | Contig4100 |
|  | NA | Contig6779 |
|  | 1,406.0 | Contig114 |
|  | 686.9 | Contig9297 |
|  | 371.9 | Contig13211 |
|  | 238.0 | Contig14754 |
|  | 183.4 | Contig13165 |
|  | 138.5 | Contig8421 |
|  | 121.0 | Contig2430 |
|  | 120.4 | Contig732 |
|  | 69.3 | Contig14950 |
|  | 50.5 | Contig8412 |
|  | 19.0 | Contig19608 |
|  | 15.2 | Contig9016 |
|  | 6.8 | Contig19148 |
|  | 1.8 | Contig6735 |
| GH11 | NA | Contig10738 |
|  | NA | Contig12100 |
|  | NA | Contig14117 |
|  | NA | Contig18110 |
|  | NA | Contig18747 |
|  | NA | Contig4024 |
|  | 4,483.0 | Contig3216 |
|  | 1,141.1 | Contig15391 |
|  | 520.9 | Contig18216 |
|  | 411.8 | Contig7007 |
|  | 262.8 | Contig15284 |
|  | 164.4 | Contig16385 |
|  | 122.0 | Contig8371 |
|  | 115.7 | Contig9069 |
|  | 50.8 | Contig17171 |
| GH13 | NA | Contig148 |
|  | 250.2 | Contig4455 |
|  | 174.4 | Contig16579 |
|  | 92.6 | Contig315 |
|  | 91.8 | Contig18827 |
|  | 82.9 | Contig4073 |
|  | 66.8 | Contig13937 |
|  | 32.4 | Contig11384 |
|  | 12.3 | Contig5000 |
|  | 6.2 | Contig16888 |
| GH15 | 12.5 | Contig17069 |
| GH16 | NA | Contig878 |
|  | 195.2 | Contig9707 |
| GH18 | 316.7 | Contig3988 |
|  | 75.4 | Contig10964 |
|  | 38.6 | Contig3393 |
|  | 37.6 | Contig8384 |
| GH19 | NA | Contig1527 |
|  | 489.9 | Contig18870 |
|  | 452.8 | Contig9503 |
|  | 343.0 | Contig19731 |
|  | 162.8 | Contig2672 |
|  | 79.2 | Contig591 |
| GH26 | NA | Contig6128 |
|  | 190.2 | Contig1498 |
|  | 81.4 | Contig327 |
|  | 26.2 | Contig3329 |
| GH31 | 271.6 | Contig13446 |
|  | 205.9 | Contig10831 |
|  | 196.1 | Contig6246 |
|  | 67.7 | Contig10885 |
|  | 34.2 | Contig19 |
|  | 18.4 | Contig17012 |
| GH32 | 67.9 | Contig1851 |
| GH38/GH57 | NA | Contig1131 |
|  | NA | Contig13466 |
|  | NA | Contig16567 |
|  | NA | Contig5169 |
|  | 843.2 | Contig8520 |
|  | 237.6 | Contig10767 |
|  | 226.3 | Contig6192 |
|  | 202.6 | Contig14132 |
|  | 186.6 | Contig16080 |
|  | 167.3 | Contig2813 |
|  | 65.4 | Contig12381 |
| GH39 | 55.0 | Contig4168 |
| GH43 | NA | Contig11636 |
|  | NA | Contig14813 |
|  | NA | Contig18200 |
|  | NA | Contig3373 |
|  | NA | Contig9354 |
|  | 1,779.3 | Contig10858 |
|  | 1,243.2 | Contig19309 |
|  | 647.5 | Contig19103 |
|  | 632.8 | Contig197 |
|  | 616.7 | Contig16438 |
|  | 602.5 | Contig12342 |
|  | 311.8 | Contig10088 |
|  | 291.0 | Contig1655 |
|  | 214.2 | Contig1253 |
|  | 170.7 | Contig3412 |
|  | 72.9 | Contig2256 |
|  | 61.1 | Contig13703 |
|  | 47.1 | Contig2151 |
|  | 27.9 | Contig11459 |
|  | 17.7 | Contig16314 |
| GH45 | NA | Contig14162 |
|  | NA | Contig16200 |
|  | NA | Contig16823 |
|  | NA | Contig19183 |
|  | NA | Contig19214 |
|  | NA | Contig19624 |
|  | NA | Contig3113 |
|  | 271.2 | Contig4134 |
|  | 226.7 | Contig7258 |
|  | 225.7 | Contig4927 |
|  | 136.9 | Contig1613 |
|  | 53.7 | Contig14086 |
|  | 28.6 | Contig19364 |
|  | 0.0 | Contig2536 |
| GH47 | NA | Contig9813 |
| GH48 | NA | Contig14201 |
|  | 6,167.8 | Contig13654 |
|  | 995.1 | Contig117 |
|  | 324.1 | Contig4845 |
|  | 282.6 | Contig13504 |
|  | 272.2 | Contig6576 |
|  | 198.4 | Contig8704 |
|  | 151.4 | Contig17001 |
|  | 130.9 | Contig1442 |
|  | 129.2 | Contig14484 |
|  | 88.1 | Contig69 |
|  | 74.7 | Contig2523 |
| GH53 | 32.1 | Contig16076 |
| GH57 | NA | Contig626 |
|  | 13.8 | Contig16520 |
